# Modulating the Electronic Structure of FeCo Nanoparticles in N‐Doped Mesoporous Carbon for Efficient Oxygen Reduction Reaction

**DOI:** 10.1002/advs.202200394

**Published:** 2022-03-24

**Authors:** Guihua Zhu, Haoyu Yang, Ying Jiang, Ziqi Sun, Xiaopeng Li, Jianping Yang, Haifeng Wang, Rujia Zou, Wan Jiang, Pengpeng Qiu, Wei Luo

**Affiliations:** ^1^ State Key Laboratory for Modification of Chemical Fibers and Polymer Materials College of Materials Science and Engineering Institute of Functional Materials Donghua University Shanghai 201620 China; ^2^ School of Chemistry and Physics Centre for Materials Science Queensland University of Technology (QUT) Brisbane QLD 4000 Australia; ^3^ Materials Genome Institute Shanghai University Shanghai 200444 P. R. China

**Keywords:** binding energy, electronic structure, FeCo nanoparticles, mesoporous carbon, oxygen reduction

## Abstract

The development of highly efficient and stable oxygen reduction electrocatalysts and revealing their underlying catalytic mechanism are crucial in expanding the applications of metal‐air batteries. Herein, an excellent FeCo alloy nanoparticles (NPs)‐decorated N‐doped mesoporous carbon electrocatalyst (FeCo/NC) for oxygen reduction reaction, prepared through the pyrolysis of a dual metal containing metal‐organic framework composite scaffold is reported. Benefiting from the highly exposed bimetal active sites and the carefully designed structure, the Fe_0.25_Co_0.75_/NC‐800 catalyst exhibits a promising electrocatalytic activity and a superior durability, better than those of the state‐of‐the‐art catalysts. Suggested by both the X‐ray absorption fine structures and the density functional theoretical calculation, the outstanding catalytic performance is originated from the synergistic effects of the bimetallic loading in NC catalysts, where the electronic modulation of the Co active sites from the nearby Fe species leads to an optimized binding strength for reaction intermediates. This work demonstrates a class of highly active nonprecious metals electrocatalysts and provides valuable insights into investigating the structure–performance relationship of transition metal‐based alloy catalysts.

## Graphical abstract

A FeCo alloy nanoparticles (NPs)‐functionalized N‐doped mesoporous carbon material is constructed and demonstrated as a promising catalyst for oxygen reduction reaction. Through modulating the electronic structure of FeCo NPs in the framework, a moderate binding energy for oxygen intermediate species is generated, thus boosting the catalytic performance.

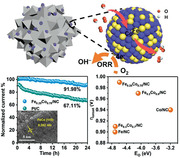

## 1 Introduction

Fuel cells and metal‐air batteries have attracted tremendous attention in the development of green energy conversion and storage devices because of their high energy density, environmental friendliness, and high safety.^[^ ^1^, ^2^, ^3^, ^4^, ^5^, ^6^ ^]^ However, the oxygen reduction reaction (ORR), a critical half‐cell reaction on the cathode side, usually suffers from sluggish kinetics owing to the multiple electron transfer pathways and reaction intermediates.^[^ ^7^ ^]^ Up to date, noble metal (such as Pt, Pd, Ru)‐based materials have been demonstrated as the state‐of‐the‐art ORR catalysts for expediting the four‐electron transfer process.^[^ ^8^, ^9^, ^10^ ^]^ Nevertheless, the formidable challenges such as poor stability, high cost, and easy poisoning severely hinder their large‐scale practical applications in these energy storage systems.^[^ ^11^, ^12^, ^13^, ^14^, ^15^ ^]^

With this aim, great efforts have been made to explore Pt substitutes, among which transition metal nanoparticles (NPs)‐functionalized N‐doped porous carbon materials (M/NC) have been regarded as one of the most promising candidates benefiting from the great synergistic effect from the porous carbon frameworks and metal centers.^[^ ^16^, ^17^, ^18^, ^19^, ^20^, ^21^, ^22^ ^]^ Sharma et al. have proved that the incorporation of cobalt NPs in graphene shell could greatly reduce the work function of latter one, which generates a lowered energy for the transfer of electrons to the adsorbed oxygen, thus resulting in an exceptionally high ORR activity and durability.^[^ ^23^ ^]^ Wang et al. have reported the synthesis of Fe NPs‐embedded porous carbon catalyst, which achieved an ORR activity with a comparable half‐wave potential (≈0.82 V vs reversible hydrogen electrode (RHE)) to that of Pt/C catalyst (≈0.84 V vs RHE).^[^ ^24^ ^]^ The high performance was attributed to the presence of Fe NPs and large surface area that could provide multiple active sites for oxygen species adsorption and transformation. Despite great success has been achieved, the performances of these single metal‐based NC catalysts are generally limited owing to the discontinuous band structure of single metal, of which the electronic structures are hard to be tuned precisely for the best case.^[^ ^25^, ^26^ ^]^ Considering that the band properties are closely related to the type and the number of metals, the construction of dual metal NPs‐decorated NC catalyst have recently attracted the most attention because the introduction of complementary dual metals could not only adjust the electronic structure in a more precise way, but also provide multiple atomic interfaces for the adsorption of oxygen‐based adsorbates and expedite ORR reaction pathways, thus leading to a remarkably enhanced electrocatalytic activity and selectivity.^[^ ^25^, ^26^, ^27^, ^28^, ^29^ ^]^ For example, Jiang et al. have reported the synthesis of FeCo alloy NPs‐embedded N, P‐codoped carbon‐coated nitrogen‐doped carbon nanotubes (NPC/FeCo@NCNTs) for an efficient bi‐functional catalysis. The enhanced catalytic activity is derived from the interplay between FeCo NPs and NCNT and the presence of N, P‐codoped carbon structure.^[^ ^30^ ^]^ Although some excellent works have been reported, the facile synthesis of a dual transition metal NPs‐functionalized NC catalyst and construction of the electronic structure‐related performance relationship remain a great challenge.

Metal‐organic frameworks (MOFs) are very promising scaffolds for the synthesis of M/NC hybrid materials due to their intrinsic properties of containing diverse organic functional groups and metal centers, which can be in situ transformed into a porous carbon framework and functional metal species followed by a carbonization process.^[^ ^31^, ^32^, ^33^ ^]^ Encouraged by these facts, we have developed a simple two‐step strategy to synthesize FeCo alloy NPs‐functionalized N‐doped mesoporous carbon materials derived from coordination‐driven self‐assembled MOF composites, which have been demonstrated as an excellent ORR electrocatalyst. Through tuning the electronic structure of FeCo NPs embedded in the composites, the catalyst displays a much better ORR performance than the commercial Pt/C catalyst including a large half‐wave potential, a high durability, and a strong tolerance toward methanol. Both the experimental results and density functional theory (DFT) calculations suggest that the introduction of neighbor Fe atoms on the Co active sites could modulate the electronic structure of the resultant alloy NPs, which generates a moderate binding energy for oxygen intermediates (e.g., *OOH and *OH), thereby significantly improving the catalytic activity and durability.

## 2 Results and Discussion

**Figure 1:**
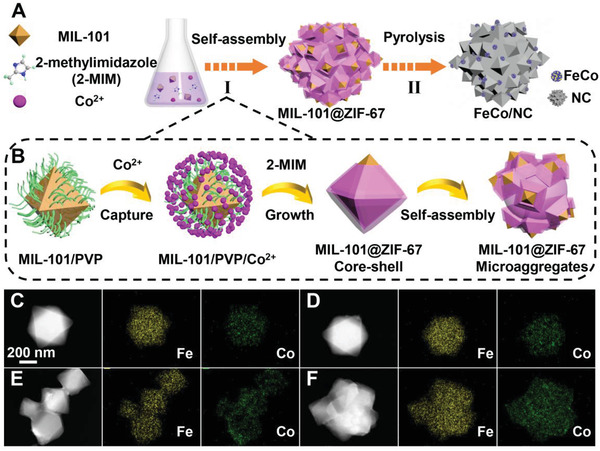
A) Schematic illustration for the preparation of the FeCo/NC catalyst. B) Schematic illustration for the coordination‐driven self‐assembly process. C–F) TEM images of intermediate product after reaction of C) 2, D) 5, E) 10, and F) 20 min.

The FeCo/NC porous nanostructures were constructed through a facile two‐step approach including the construction of a dual‐metal containing MIL‐101@ZIF‐67 MOF superstructure, followed by a pyrolysis process (**Figure** **1**A). In the first step, a novel coordination‐driven self‐assembly strategy was developed to synthesize the MOF scaffold by using the polyvinylpyrrolidone (PVP)‐modified MIL‐101 nanocrystals (NCs) as the building blocks (Figure 1B). The appearance of C═O resonance peak at 1657 cm^–1^ in the Fourier transform infrared (FTIR) spectrum of MIL‐101/PVP suggests that the PVP molecules were successfully grafted on the surface of MIL‐101^[^ ^34^ ^]^ (Figure S1, Supporting Information). Note that the introduction of PVP ligands is very important for the further assembly because they contain abundant nitrogen and oxygen‐containing groups, which could capture the cobalt ions in the solution on the surfaces of MIL‐101. Therefore, when another ligand 2‐methylimidazole (2‐MIM) was added into the reaction system, the shell layer of ZIF‐67 crystals could grow on the MIL‐101 seeds due to the strong coordination effect between the cobalt ion and 2‐MIM. In the presence of excess ZIF‐67 forming precursors, such coordination and linkage would induce the further self‐assembly of MIL‐101, thus forming a multicore–shell‐structured MIL‐101@ZIF‐67 composites. Without the decoration of PVP ligands on the MIL‐101 NCs, separated MIL‐101 and ZIF‐67 structures were obtained (Figure S2, Supporting Information). To confirm the mechanism, the time‐dependent experiments were carried out. The dark‐field transmission electron microscopy (TEM) images and the corresponding elemental maps (Figure 1C,D) clearly show that the distribution of cobalt ions on MIL‐101 gradually increased as rising the reaction time from 2 to 5 min. When the reaction time was prolonged to 10 min, a well‐defined core–shell‐structured MIL‐101@ZIF‐67 was formed (Figure 1E). Further increasing the reaction time could lead to the spontaneous assembly of MIL‐101@ZIF‐67 into large microaggregates (Figure 1F). When the precursors were exhausted, multicore–shell‐structured MIL‐101@ZIF‐67 composites with MIL‐101 NCs mounted on the ZIF‐67 framework were obtained. In addition, both the scanning electron microscope (SEM) and TEM images (Figures S3 and S4, Supporting Information) show that the size of MIL‐101 gradually increased and finally assembled into large aggregates with the increase of the reaction time, further confirming the claim. In the zeta potential test, the positive charge decreased after adding 2‐methylimidazole into the MIL‐101/PVP@Co^2+^ complex, indicating the weak electrostatic interaction generated during the coordination‐induced assembly process (Figure S5, Supporting Information). In the final carbonization step, the binding ligands serve as the nitrogen and carbon source while the metal ions (Fe^2+^ and Co^2+^) serve as metal precursors, resulting in the formation of the dual metal‐containing nitrogen‐doped carbon framework. Simultaneously, under the assistance of redox conditions generated during pyrolysis, metal ions could be reduced to FeCo NPs. Finally, a composite electrocatalyst consisted of uniformly dispersed FeCo NPs in a porous nitrogen‐doped carbon framework was obtained. Several samples with different Fe/Co ratios (*x*) and carbonization temperature (*y*) were prepared and denoted as Fe*_x_* Co_1−_ *_x_*/NC‐*y*. The content of metals was examined by the inductively coupled plasma optical emission spectrometry (ICP‐OES, Table S1, Supporting Information), matching well with the precursor adding ratio.

**Figure 2:**
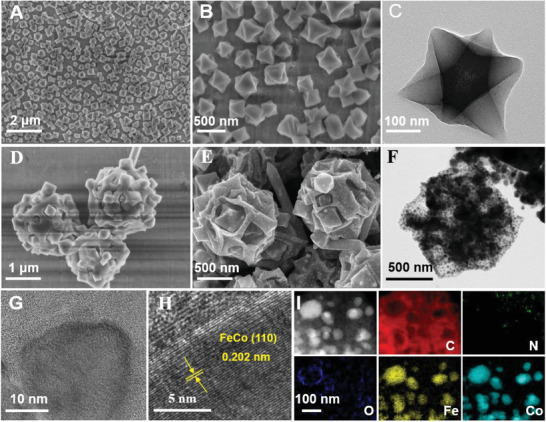
A,B) SEM images and C) TEM image of PVP‐modified MIL‐101, SEM images of D) MIL‐101@ZIF‐67 as‐made sample and E) Fe_0.25_Co_0.75_/NC‐800, F) TEM image of Fe_0.25_Co_0.75_/NC‐800, and G,H) HRTEM, and I) high‐angle annular dark‐field scanning TEM (HAADF‐STEM) images and corresponding C, N, O, Fe, and Co elemental mappings for Fe_0.25_Co_0.75_/NC‐800.

The microstructure and morphology of the resultant catalysts were further investigated by TEM and SEM observation. The PVP‐modified MIL‐101 seeds possess a uniform octahedral structure with an average particle size of ≈250 nm (**Figure** **2**A–C). After the coordination‐driven self‐assembly process, the multicore–shell‐structured MIL‐101@ZIF‐67 composite with a particle size of ≈2 µm was achieved, exhibiting a rough surface composed of randomly mounted MIL‐101 NCs (Figure 2D and Figure S6, Supporting Information). The powder X‐ray diffraction (XRD) pattern of MIL‐101@ZIF‐67 composite MOF possesses the characteristic peaks from both the MIL‐101 and ZIF‐67 crystallites, further suggesting the formation of the composites (Figure S7, Supporting Information). Moreover, the surface morphology of the MOF scaffolds can be finely tuned by varying the concentration of MIL‐101 in the assembly process (Figures S8–S10, Supporting Information). At the same time, the particle size of the scaffold could be varied from 1.6 to 2.5 µm. After being subjected to a pyrolysis process, the FeCo/NC composites were formed with uniform NPs in a diameter of ≈30 nm distributed on their surfaces (Figure 2E–G and Figures S8–S10, Supporting Information). The high‐resolution TEM (HRTEM) image of Fe_0.25_Co_0.75_/NC‐800 shows the lattice spacing of ≈0.202 nm, which matches well with the (110) lattice fringe of the FeCo alloy NPs^[^ ^35^, ^36^ ^]^ (Figure 2H). The electron energy loss spectroscopy (EELS) element mapping and energy‐dispersive X‐ray spectroscopy (EDS) mapping images of the FeCo/NC composites reveal the existence of Fe, Co, C, N, and O elements in all the pyrolysis products. In addition, the distribution of Fe and Co elements were overlapped, further indicating the formation of FeCo alloy NPs (Figure 2I and Figures S8–S10, Supporting Information). As a comparison, the monometallic NPs‐functionalized NC framework (Co/NC‐800 and Fe/NC‐800) was also prepared (Figures S11 and S12, Supporting Information).

**Figure 3:**
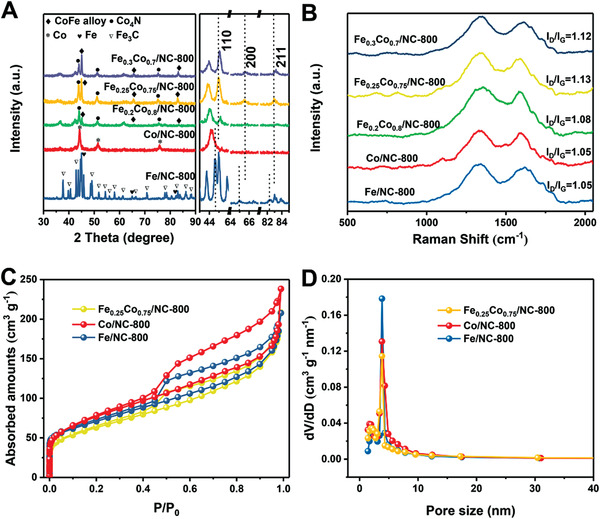
A) XRD analysis and B) Raman spectra of the as‐synthesized electrocatalysts under 800 ℃ pyrolysis. C) N_2_ adsorption–desorption isotherm and (D) corresponding BJH pore size distribution of the Fe/NC‐800, Co/NC‐800, and Fe_0.25_Co_0.75_/NC‐800 electrocatalyst.

The crystallographic structures of the FeCo alloy NPs in the resultant materials were further determined by the powder XRD analysis. It is clear that the dominant phase is bimetallic FeCo alloy with several sharp characteristic peaks centered at nearly 44.87°, 65.31°, and 82.74°, which can be well indexed to the (110), (200), and (211) crystal planes of body centered cubic (BCC) FeCo alloys (JCPDS no. 49–1568) (**Figure** **3**A). In the enlarged plots, the three major peaks (110), (200), and (211) were slightly shifted to a larger angle compared with the single metal‐decorated sample, possibly owing to the lattice contraction of the crystal structure after the incorporation of iron atoms.^[^ ^25^, ^37^ ^]^ The weak diffraction peak at 43.71° corresponding to the Co_4_N phase (JCPDS no. 41–0943) was also detected in the FeCo functionalized samples and its intensity increased as rising the calcination temperature from 700 to 900 °C (Figure S13, Supporting Information).^[^ ^37^ ^]^ This is possibly due to the release of NH_3_ from the decomposition of the imidazole ring at a higher carbonization temperature, which promotes the formation of Co_4_N phase.^[^ ^37^, ^38^ ^]^ Dai et al. demonstrated the synergistic effect of FeCo alloy NPs and Co_4_N within a porous N‐doped carbon (N—C) framework^[^ ^37^ ^]^ Zhang et al. combined metallic Co_4_N and Co—N—C for flexible Zn–air batteries.^[^ ^38^ ^]^ All of these results proved that the simultaneous installation of multiple cobalt‐based active phases, including FeCo alloys and Co_4_N NPs, plays a critical role in promoting the ORR performance.

The Raman spectra of resultant catalysts show two peaks at 1340 cm^−1^ (D‐band) and 1588 cm^−1^ (G‐band), corresponding to sp^3^ disordered carbon and sp^2^ graphitic carbon, respectively^[^ ^39^ ^]^ (Figure 3B). The intensity ratio (*I*_D_/*I*_G_) indicates the graphitization degree and defect density of carbon‐based materials. The *I*_D_/*I*_G_ values were calculated to be 1.05, 1.05, 1.08, 1.13, and 1.12 for the Fe/NC‐800, Co/NC‐800, Fe_0.2_Co_0.8_/NC‐800, Fe_0.25_Co_0.75_/NC‐800, and Fe_0.3_Co_0.7_/NC‐800 electrocatalysts, respectively, revealing that the encapsulation of alloy NPs leads to more defect sites in the carbon matrix.^[^ ^40^, ^41^ ^]^ The ORR performance is in association with the Brunauer–Emmett–Teller (BET) surface area and pore properties of the catalyst. Therefore, nitrogen adsorption–desorption isotherms were examined, displaying a typical type IV curve with a H_2_‐type hysteresis loop, in reflection of the existence of mesopores^[^ ^42^, ^43^, ^44^ ^]^ (Figure 3C). The pore size of the Fe_0.25_Co_0.75_/NC‐800 composites calculated from the adsorption data using the Barrett–Joyner–Halenda (BJH) model was ≈3.83 nm (Figure 3D). In addition, the BET surface area and pore volume were calculated to be 223.53 m^2^ g^−1^ and 0.32 cm^3^ g^−1^, respectively, which could provide abundant actives sites for ORR (Table S2, Supporting Information).^[^ ^45^, ^46^, ^47^, ^48^ ^]^ The BET surface area of alloy NPs‐decorated carbon materials is slightly smaller than those of single‐metal NPs‐decorated samples, possibly owing to the larger particle size of the former one. The thermogravimetric analysis (TGA) infers that the total metal content of Co/NC‐800, Fe/NC‐800, and Fe_0.25_Co_0.75_/NC‐800 electrocatalysts are 64.96, 64.89, and 66.01 wt%, respectively (Figure S14, Supporting Information), in good agreement with the ICP results.

**Figure 4:**
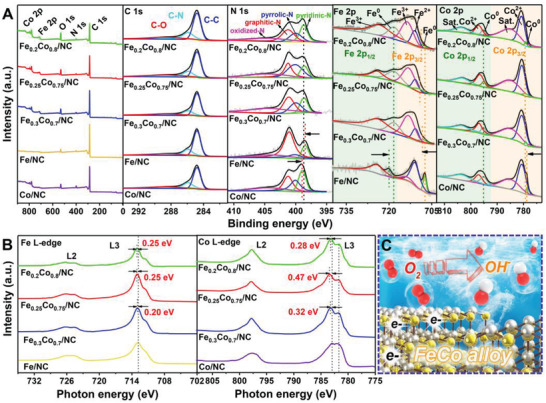
A) XPS characterizations of Fe_0.2_Co_0.8_/NC‐800, Fe_0.25_Co_0.75_/NC‐800, Fe_0.3_Co_0.7_/NC‐800, Fe/NC‐800, and Co/NC‐800 electrocatalyst including the total survey spectra, and the dissociated spectra of C 1s, N 1s, Fe 2p, and Co 2p. B) Comparison of Fe L‐edge and Co L‐edge XANES in different electrocatalyst samples. C) Schematic illustration for the ORR catalytic mechanism of the electrocatalyst.

The surface chemical states of the mono‐ and bimetallic NPs‐functionalized carbon matrix were performed by high‐resolution X‐ray photoelectron spectroscopy (XPS) analysis (**Figure** **4**A and Table S3, Supporting Information). The C 1s region of XPS in all the samples can be dissociated into three types of carbon species at 284.8, 285.9, and 288.5 eV, corresponding to C—C, C—N, and C—O species, respectively. The detection of C—N for the catalysts indicates that N atoms were successfully doped into the carbon materials.^[^ ^35^ ^]^ The high‐resolution N 1s spectra (Figure 4A and Table S4, Supporting Information) display four contributions of nitrogen species, involving pyridinic‐N, graphitic‐N, pyrrolic‐N, and oxidic‐N, located at 398.6, 400.1, 401.2, and 404.5 eV, respectively. The presence of high content of pyridinic‐N and pyrrolic‐N contributes the excellent ORR performance.^[^ ^49^ ^]^ Moreover, the peak of pyridinic‐N was clearly shifted after forming alloys, revealing the electronic interaction between the metal and nitrogen atoms. Compared with the Co/NC sample, the introduction of the Fe species resulted in the shift of pyridinic‐N peak to a lower energy level (shifted right in the figure), suggesting that N atoms obtained more electrons, which are originated from the Fe atoms. On the contrary, the introduction of Co atoms in the Fe/NC sample caused the shift of pyridinic‐N to a high energy level (shifted left in the figure), indicating that the pyridinic‐N donated electrons to the Co atoms. In this perspective, the Fe atoms act as the electron reservoir that donates electrons to the system while Co as the electron depletion center that accepts electrons. The Fe_0.25_Co_0.75_/NC‐800 electrocatalyst exhibits two signals at 790.4 and 718.2 eV in Fe 2p spectrum, corresponding to zero‐valence state Fe, which is derived from FeCo alloy.^[^ ^29^, ^49^ ^]^ The Fe 2p_3/2_ signal can be decomposed in two contributions of Fe^2+^ and Fe^3+^ species at 710.7 and 713.1 eV, respectively. Notably, all bimetallic alloy samples present a positive shift of the Fe^0^ peak in Fe 2p_3/2_ orbitals and a negative shift of the Fe^0^ peak in Fe 2p_1/2_ orbitals compared with those of the Fe/NC catalyst, confirming a change in the electronic structure after forming the alloy NPs.^[^ ^25^, ^26^ ^]^ The Co 2p spectra of Fe_0.25_Co_0.75_/NC‐800 were deconvoluted into two cobalt species, being assigned to Co^0^ (779.1 and 795.1 eV) and Co^2+^ (780.6 and 796.5 eV), respectively.^[^ ^29^, ^37^ ^,50]^ Under the influence of neighbor Fe atoms, the binding energy for Co^0^ was downward shifted, further suggesting the change in the electronic structure of FeCo NPs. While the significant charge redistribution around FeCo metal centers might provide a favorable surface energy, which benefits the adsorption/desorption process of oxygen species on the surface of alloy NPs, thus boosting the overall rates of interfacial catalytic processes.^[^ ^38^, ^51^ ^]^ The electronic structure change of FeCo alloy NPs in the catalyst was further investigated by X‐ray near‐edge structure (XANES) analysis (Figure 4B). L‐edge XANES involves the excitation of 2p core electrons into partially unoccupied 3d states, which provides direct evidence of charge transfer between Fe and Co. At Fe L_3_ edge, two groups of peaks including a strong peak at 713.13 eV and a weak shoulder peak at 711.49 eV were detected, corresponding to the chemical state of Fe^3+^ and Fe^2+^, respectively (Figure 4B). In contrast to the single Fe NPs‐decorated NC catalyst, the binding energy of Fe^3+^ in Fe_0.25_Co_0.75_/NC was slightly shifted left (≈0.25 eV) due to the ligand field effects of Fe and Co.^[^ ^52^ ^]^ At the Co L_3_ edge, more clear electronic structure differences were observed (Figure 4B). The distinct peaks at 783.48 and 781.68 eV can be assigned to the Co^3+^ ions at octahedral sites and Co^2+^ ions at tetrahedral sites, respectively.^[^ ^53^ ^]^ The peak intensity ratio of Co^3+^/Co^2+^ for Fe_0.25_Co_0.75_/NC increased significantly compared with that of Co/NC, suggesting that the participation of Fe in the composite can modulate the electronic state of Co to obtain a higher oxidative state, which is conducive to the ORR performance.^[^ ^53^, ^54^ ^]^ This matches well with the previous result, reported by Wang et al., showing that Co^3+^ at octahedral sites can offer mediated adsorption ability to the reactive oxygen intermediates in ORR process.^[^ ^55^ ^]^ In addition, Co^3+^ in Co 2p 3/2 at 783.48 eV exhibited 0.47 eV left shifts to that of Co/NC (783.01 eV), also suggesting the interaction between Fe and Co atoms. Through analyzing the fine structure and chemical valence state of FeCo/NC, we can conclude that the reconstruction of electronic structure through FeCo alloying can effectively adjust the adsorption energy of ORR intermediates, which may significantly affect the ORR performance of FeCo/NC electrocatalyst (Figure 4C).

**Figure 5:**
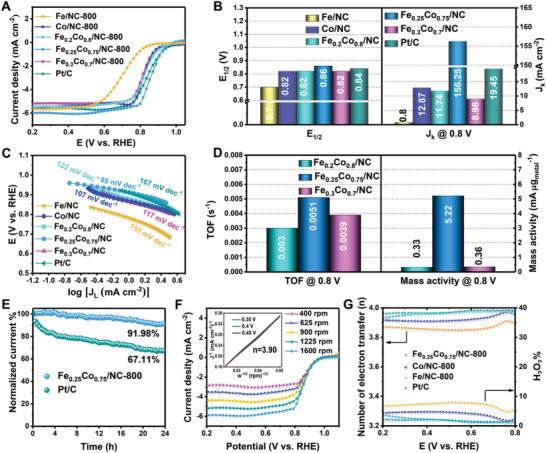
A) ORR polarization curves. B) *E*_1/2_ and *J*_K_ at 0.8 V and C) corresponding Tafel slopes for all as‐synthesized electrocatalysts under 800 ℃ pyrolysis, and commercial Pt/C in O_2_‐saturated 0.1 m KOH at 0.01 V s^−1^ under a rotating speed of 1600 rpm. D) Comparison of the TOFs and mass activity of Fe_0.2_Co_0.8_/NC, Fe_0.25_Co_0.75_/NC, and Fe_0.3_Co_0.7_/NC. E) Chronoamperometric response at 0.6 V (vs RHE) for 24 h. F) LSV curves of Fe_0.25_Co_0.75_/NC‐800 at various speeds at a scan rate of 0.01 V s^−1^; inset is corresponding Koutecky–Levich plots at different potentials. G) Electron transfer number (*n*) and H_2_O_2_ yield of electrocatalysts calculated by RRDE curves from 0.2 to 0.8 V (vs RHE).

The electrocatalytic activity of resultant catalysts was investigated by rotating disk electrode (RDE) measurements in O_2_‐saturated 0.1 m KOH solution. The CV curves of both mono‐ and bimetallic catalysts in saturated O_2_ solution show a well‐defined ORR cathodic peak, revealing the presence of oxygen reduction property. Obviously, bimetallic Fe_0.25_Co_0.75_/NC‐800 samples exhibit a more positive cathodic peak (0.78 V) than other comparison samples, indicating a better electrocatalytic ORR performance (Figure S15, Supporting Information). The ORR polarization curves of the as‐synthesized electrocatalysts (**Figure** **5**A,B) show that the Fe_0.25_Co_0.75_/NC‐800 catalyst exhibits the best performance including a high half‐wave potential (0.86 V vs RHE), a positive onset potential (0.99 V vs RHE), and a large limiting current density (6.01 mA cm^–2^), which is much better than the monometallic catalyst, bimetallic catalysts with other ratios, and even the Pt/C catalyst. Furthermore, the kinetic current density (*J*_k_) at 0.8 V reveals that Fe_0.25_Co_0.75_/NC (156.25 mA cm^−2^) is larger than that of Fe_0.2_Co_0.8_/NC (11.74 mA cm^−2^), Fe_0.3_Co_0.7_/NC (8.98 mA cm^−2^), and Pt/C (19.45 mA cm^−2^), indicating a faster reaction kinetics and effective pervasion of reactants for Fe_0.25_Co_0.75_/NC electrocatalysts (Figure 5B). In addition, the Tafel slope of Fe_0.25_Co_0.75_/NC catalyst (Figure 5C) is calculated to be 88 mV dec^− 1^, much lower than the comparison samples, implying that the Fe_0.25_Co_0.75_/NC‐800 composites possess an excellent ORR catalytic kinetic. These results suggest that the ORR catalytic performance of the bimetallic systems catalysts have a strong composition dependence. The linear sweep voltammetry (LSV) curves of Fe_0.25_Co_0.75_/NC sample carbonized at different temperature (700, 800, and 900 ℃) were also examined (Figure S16, Supporting Information), revealing that middle 800 ℃ is the optimal temperature. This can be explained by that increasing the pyrolysis temperature can significantly enhance the electrical conductivity of the electrocatalyst and further promote the catalytic activity while too high temperature could also lead to a sharp contraction of the MOF composites architecture, which causes the falling off of the surface MIL‐101, thus resulting in a poor ORR catalytic performance (Figure S17, Supporting Information). In addition, the calcined FeCo/NC sample derived from MIL‐101@ZIF‐67 without PVP functionalization exhibits significantly degraded ORR performance due to the separation of MIL‐101 and ZIF‐67 (Figure S18, Supporting Information). The turnover frequency (TOF) was calculated at 0.8 V versus RHE to evaluate the intrinsic activity of the FeCo/NC with different FeCo ratio, which assumed that all of the Fe and Co atoms acted as active sites in the samples. The TOF value of 0.0051 s^−1^ for Fe_0.25_Co_0.75_/NC indicates a higher active site utilization efficiency than in Fe_0.2_Co_0.8_/NC and Fe_0.3_Co_0.7_/NC, which may be ascribed to the surface electronic structure reconfiguration that resulted in an adjustment of adsorption energy for ORR intermediates (Figure 5D). To gain a better understanding of the observed catalytic activities of the produced FeCo electrocatalysts for ORR, the mass activities at 0.80 V were calculated based on the *J*_k_ and the amount of alloy metal on the electrode (Figure 5D). The mass activity of Fe_0.25_Co_0.75_/NC‐800 is calculated to be 5.22 mA μg_metal_^−1^, which is 15.8 and 14.5 times larger than those of the Fe_0.2_Co_0.8_/NC‐800 (0.33 mA μg_metal_^−1^) and Fe_0.3_Co_0.7_/NC‐800 (0.36 mA μg_metal_^−1^) catalysts, respectively.

The stability tests of the Fe_0.25_Co_0.75_/NC‐800 and Pt/C catalysts were further implemented via chronoamperometric analysis (Figure 5E). The Fe_0.25_Co_0.75_/NC‐800 maintains ≈92% of the original current density after continuous testing for 24 h, whereas the commercial Pt/C catalyst only retains 67%, indicating the superior durability of Fe_0.25_Co_0.75_/NC‐800 catalyst. Moreover, the tolerance of ORR catalysts against some poisoning contaminants (e.g., methanol) plays an important role in the practical application. Therefore, the methanol durability of Fe_0.25_Co_0.75_/NC‐800 and Pt/C catalyst was also investigated through the chronoamperometric responses after the introduction of 1 m methanol (Figure S19, Supporting Information). The current density of the Fe_0.25_Co_0.75_/NC‐800 catalyst slightly increases, whereas Pt/C displays a remarkable reduction in the current density under the same conditions, verifying that the Fe_0.25_Co_0.75_/NC‐800 catalyst has a strong tolerance to methanol. The increase of the current density here is possibly due to the sudden change of the concentration of reactive species after addition of massive volume of methanol. More importantly, the comprehensive ORR performances of the Fe_0.25_Co_0.75_/NC‐800 catalyst are much better than those of alloy NPs‐functionalized electrocatalysts reported recently (Table S5, Supporting Information).

In the process of ORR, multi‐step electron transfer pathways are involved, which generate multiple reaction intermediates such as O*, OH*, and OOH* (Step 1 to 4). To clarify the catalytic mechanism, two chemical descriptors including number of electron transferred (*n*) and oxygen binding energy (*E*_O_) are generally used. Here, the *n* was determined by the Koutecky–Levich (K–L) plots and rotating ring‐disk electrode (RRDE) technique

(1) $$\begin{array}{ccc} & & \mathrm{Step}\;1:\;{}^{*}+O_{2}+H_{2}O+e^{-}\to {}^{*}\mathrm{OOH}+{\mathrm{OH}}^{-}\;\left(\Delta G1\right)\\ & & \mathrm{Step}\;2:\;{}^{*}\mathrm{OOH}+e^{-}\to {}^{*}O+{}^{*}{\mathrm{OH}}^{-}\;\;\;\;\;\;\left(\Delta G2\right)\\ & & \mathrm{Step}\;3:\;{}^{*}O+H_{2}O+e^{-}\to {}^{*}\mathrm{OH}+{\mathrm{OH}}^{-}\;\;\;\;\left(\Delta G3\right)\\ & & \mathrm{Step}\;4:\;{}^{*}\mathrm{OH}+e^{-}\to {}^{*}+{\mathrm{OH}}^{-}\;\;\;\;\;\;\;\;\left(\Delta G4\right) \end{array}$$

The K–L plots calculated according to the polarization curves at various rotation speeds (400–1600 rpm) show clear linearity from 0.3 to 0.5 V (Figure 5F), implying a first‐order reaction kinetic of the Fe_0.25_Co_0.75_/NC‐800 catalyst for ORR. In addition, the corresponding *n* is estimated to be 3.9, indicating that oxygen was reduced to water primarily through an ideal four‐electron reduction pathway. According to the current collected from the disk and ring, the *n* of Fe_0.25_Co_0.75_/NC is calculated to be 4, while the corresponding H_2_O_2_ yield is less than < 5%, which is close to that of commercial Pt/C catalyst (Figure 5G and Figure S20, Supporting Information), further suggesting a four‐electron transfer pathway.

**Figure 6:**
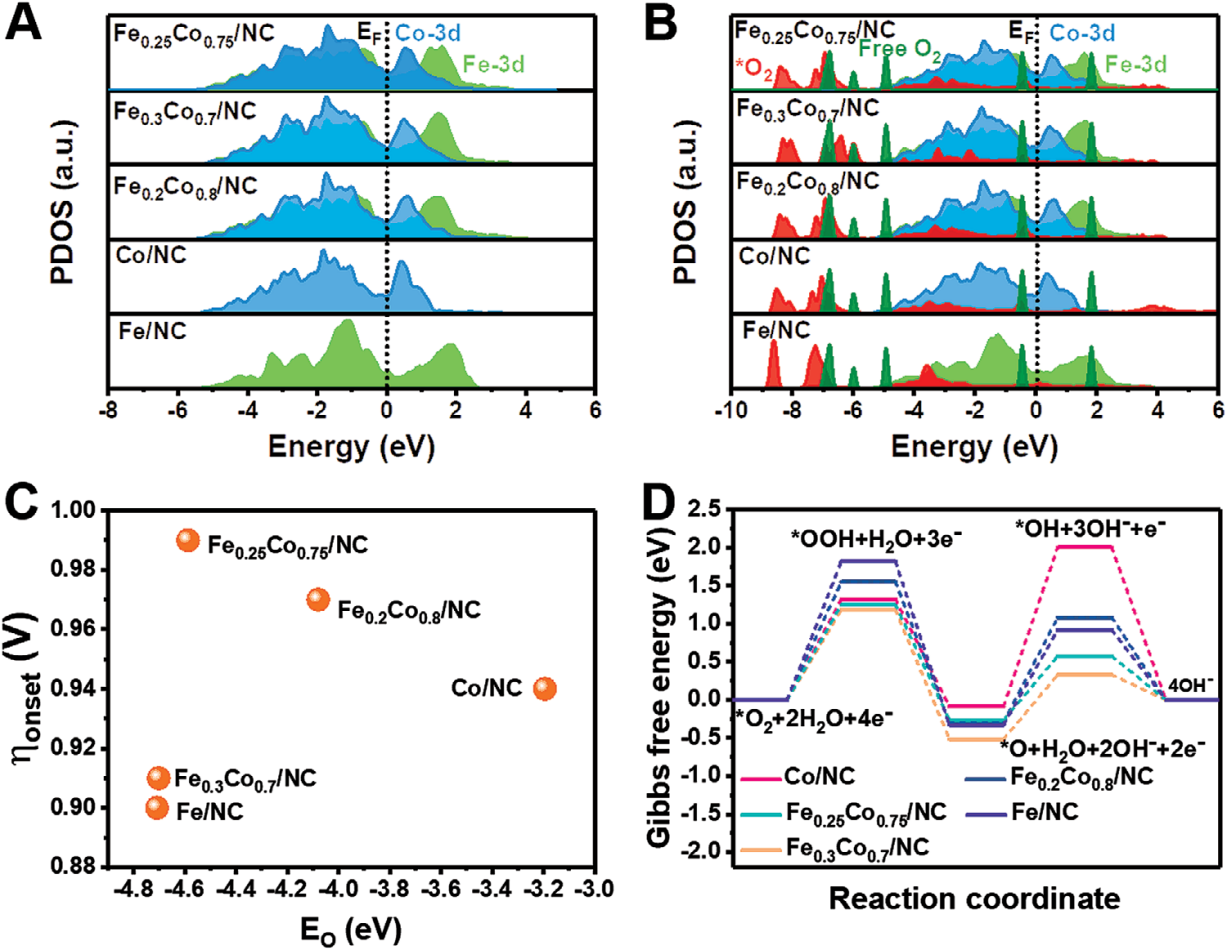
DFT studies of catalysts. A) The PDOSs of the clean FeCo/NC catalysts. B) The PDOSs for the oxygen adsorption. C) ORR onset potential as a function of the DFT‐calculated *E*_O_. D) Free energy diagrams with *U* = 0.385 V for ORR process over Fe/NC, Co/NC, Fe_0.2_Co_0.8_/NC, Fe_0.25_Co_0.75_/NC, and Fe_0.3_Co_0.7_/NC electrocatalysts.

Based on the Sabatier rule, an optimized ORR activity requires a moderate *E*_O_ value to balance both the surface oxidation and desorption process. This is because very strong binding energies could result in surface oxidation, which decreases the catalytic activity, while very weak binding energies contribute to a fast desorption of the oxygen intermediates from the active sites, limiting the oxygen reduction process.^[^ ^25^ ^]^ Therefore, DFT calculations are further performed to reveal the mechanism of Fe*_x_* Co_1−_ *_x_*/NC electrocatalysts for enhanced ORR catalytic activity. Figure S21 (left) in the Supporting Information presents the model structure of the pristine Co/NC, Fe/NC, and FeCo/NC with different ratios. It is clear that the alloying led to two different atom sites on the surface of the catalyst and disorder of atomic arrangement becomes more significant with increasing Fe atoms concentration, resulting in a higher degree of lattice distortion. The partial density of states (PDOS) was also calculated to examine the electronic structure change of the crystals (**Figure** **6**A). The results showed that the electronic structure of both Fe and Co were shifted close to the Fermi level after alloying, suggesting the strong charge redistribution between Fe and Co atoms, which matches well with the XPS and XANES analysis. For the ORR process, the initial adsorption of O_2_ molecules determines the efficiency of O_2_ reduction and the following electron transfer. The evident downshift of s,p orbitals in O_2_ has been observed after putting them on the crystal surface, which confirms the active electron transfer from the alloy to the O_2_ to achieve the stable adsorption (Figure 6B). Interestingly, the downshift increased as the Fe content in the alloys increases, suggesting that the Fe atoms act as the electron donor in the system. To better understand the ORR process on the alloy, the adoption model of intermediates (*O_2_, *OOH, *O, and *OH) on FeCo system were also proposed (Figure S21 (right), Supporting Information). From the models, the Fe*_x_* Co_1−_ *_x_*/NC electrocatalysts show the active sites mainly localized at fivefold hollow sites containing three Fe atoms and two Co atoms for a high ORR catalytic activity. According to the volcano diagram of catalytic activity and DFT‐calculated *E*_O_ values (Figure 6C), Fe_0.25_Co_0.75_/NC electrocatalyst exhibits the highest catalytic efficiency, but possesses a moderate *E*_O_ value, matching well with the experimental results. Moreover, the standard free energy diagrams with *U* = 0.385 V for ORR over Fe/NC, Co/NC, Fe_0.2_Co_0.8_/NC, Fe_0.25_Co_0.75_/NC, and Fe_0.3_Co_0.7_/NC are compared to investigate the influence of surface electronic structure on the ORR intermediates (*OH, *O, and *OOH) (Figure 6D). The maximum positive free energy change (Δ*G*) indicates the sluggish rate‐determining step. As revealed in Figure 6D, the rate‐determining step is stage 1 for Fe_0.2_Co_0.8_/NC, Fe_0.25_Co_0.75_/NC and Fe_0.3_Co_0.7_/NC, and Fe/NC, in which the *OOH formation Δ*G*1 is calculated to be 1.55, 1.18, 1.25, and 1.82 eV, respectively. The Gibbs free energy barrier of the rate‐determining step in ORR process for all FeCo/NC catalyst decreases compared with monometallic counterparts, which attributes to the strong changes in electronic structure after the incorporation of Fe atoms in the Co crystal lattice, in good agreement with experimental results. All these results clearly demonstrate that the enhanced ORR activity for FeCo/NC composites is originated from the optimized electronic structure of FeCo NPs tuned by charge redistribution. Besides the effect of the optimized electronic structure, the nitrogen‐doped mesoporous carbon support could not only serve as an efficient conductive network for the electron transport, but also provide additional active sites for the adsorption of oxygen intermediates through nitrogen doping, which is also the key to an excellent ORR performance.

## 3 Conclusion

In conclusion, we have synthesized a series of hierarchically MOF composites‐derived FeCo alloy‐functionalized mesoporous carbon materials through a simple two‐step approach involving the coordination‐driven self‐assembly of MOF composites, followed by a high‐temperature carbonization process. The Fe_0.25_Co_0.75_/NC‐800 catalyst with a large BET surface area, uniform distribution of FeCo alloy NPs, and an optimized binding energy for oxygen species, displayed excellent electrocatalytic performance toward the ORR. DFT calculations show that the active sites mainly localized at Co neighboring Fe sites are responsible for the enhanced ORR catalytic activity. The bimetallic FeCo/NC electrocatalysts showed decreased Gibbs free energies during ORR reaction compared to their monometallic counterparts, which were attributed to the substantial changes of the surface charge distribution and electronic properties. This work provides in‐depth insights into directly correlating the structure–performance relationships of FeCo NPs supported on N‐doped mesoporous carbon materials for ORR process, thereby paving an important way toward the rational construction of low‐cost and high‐performance platinum‐group‐metals‐free electrocatalysts.

## 4 Experimental Section

### Materials

Ferric chloride hexahydrate (FeCl_3_⋅6H_2_O, AR, 99%), 2‐aminoterephthalic acid (99%), cobalt nitrate hexahydrate (Co(NO_3_)_2_⋅6H_2_O, AR, 99%), 2‐methylimidazole (2‐MIM, 98%), *N*,*N*‐dimethylformamide (DMF, AR, 99.5%) were obtained from Aladdin Industrial Corporation. The commercial Pt/C catalyst (20 wt%), Nafion solution (≈5 wt%), and potassium hydroxide (metal basis, 99.99%) were obtained from Sigma‐Aldrich Co., LTD. All chemicals were used without further purification.

### Preparation of PVP‐Modified MIL‐101 MOF

The MIL‐101 MOF was fabricated through a hydrothermal method according to the literature reported previously.^[^ ^56^ ^]^ First, 0.29 g of FeCl_3_⋅6H_2_O was dissolved in 40 mL of DMF and refluxed at 160 °C for 15 min in a three‐necked flask (100 mL). Then, 0.2 g of 2‐aminoterephthalic acid was dissolved in 10 mL of DMF, and then added dropwise to the solution above. The mixed dispersion was further stirred for another 10 min. After the solution was cooled down to room temperature, the precipitates were obtained by centrifugation and washed for three times with ethanol. Subsequently, the obtained MIL‐101 MOF was dispersed in 10 mL of 5% PVP methanol solution (*m*_w_ = 5000) and kept under stirring for 12 h at ambient temperature. At last, the PVP‐functionalized MIL‐101 was collected and then dispersed in 15 mL methanol for further use.

### Preparation of MIL‐101@ZIF‐67 Composite MOF

The MIL‐101@ZIF‐67 composite MOF materials were achieved by using PVP‐functionalized MIL‐101 as the nanoseed. In a typical synthesis, 1 mL of PVP‐functionalized MIL‐101 methanol solution was added into 5 mL of methanol and then Co(NO_3_)_2_ was added (29.3 mg) under ultrasound at room temperature until homogenous solution was formed. Then, 5 mL of 2‐methylimidazole/methanol solution (64.9 mg) was quickly added into the mixed dispersion. Next, the mixed dispersion was kept standing for 4 h at ambient temperature. At last, the sample was obtained after centrifugation, washed with methanol several times, and dried in a vacuum oven at 60 ℃ for 12 h. The resultant precursor was termed as MIL‐101@ZIF‐67. The other two comparative composites were assembled with 0.75 and 1.25 mL MIL‐101 solution, respectively. As a comparison, the ZIF‐67 MOF was prepared following the same procedure without adding MIL‐101 in the synthesis.

### Preparation of FeCo/NC‐800

The FeCo/NC materials were prepared by placing MIL‐101@ZIF‐67 in a ceramic boat and heated at 800 ℃ for 2 h in a tube furnace under flowing N_2_ with the heating rate of 5 °C min^−1^. The resultant product was denoted as Fe*_x_* Co_1−_ *_x_*/NC‐800, where *x* and 1−*x* in the sample abbreviation referred to the measured molar ratio of Fe/Co in the as‐synthesized catalysts, as well as, the number represented pyrolysis temperature. For comparison, Fe/NC and Co/NC monometallic electrocatalysts were synthesized by carbonization of MIL‐101 and ZIF‐67 at 800 ℃ for 2 h, respectively.

### Characterization

The morphological structure of the catalyst was examined by field‐ emission SEM (TESCAN/MAIA3, Czech), TEM (JEM‐2100F, Talos F200S), and a Cs‐corrected TEM (Themis ETEM, Thermo Fisher Scientific) with EELS (Enfinium ER Model 977), operating at 300 kV. The EDS element mapping was performed on an FEI Talos F200S. XRD spectra were recorded on a Rigaku D/Max‐2550 PC diffractometer (Tokyo, Japan) with a Cu K*α* radiation as the X‐ray source. TGA was examined with a 209F1 Libra instrument at a heating rate of 10 ℃ min^−1^ under aerobic conditions (flow rate of 20 mL min^–1^). Raman spectra were performed with in via reflex Raman microscope by using a 532 nm laser. Metal ratio was carried out by ICP‐OES (Prodigy‐ICP). XPS spectra were determined with Escalab 250Xi equipped with Al K*α* radiation. Nitrogen sorption isotherms were performed by Quantachrome Autosorb‐iQ at 77 K after degassing in vacuum at 180 °C for more than 8 h. BJH model was used to determine the pore size distribution and pore volume. BET model was used to measure the specific surface area of catalysts. FTIR spectrum was carried out with a NEXUS‐670 instrument. The synchrotron radiation‐based XANES measurements were carried out in BL002 station in Shanghai Synchrotron Radiation Facility (SSRF).

### Electrochemical Measurements

Princeton electrochemical workstation was used to examine the ORR performance of the catalyst. Glassy carbon (5 mm diameter, area of 0.19625 cm^2^), Pt, and Ag/AgCl (saturated with 3 m KCl) were employed as the working, counter, and reference electrode, respectively. A homogenous suspension ink was synthetized as follows: 5 mg of the sample was dispersed in ethanol (475 µL) and Nafion (25 µL), and then sonicated for 1 h. Then, 6 µL of catalyst ink was carefully dropped onto the glassy carbon RDE. For comparison, 20 wt% Pt/C (Alfa Aesar) ink was prepared following the similar procedure. The cyclic voltammetry (CV) curves were examined in O_2_‐saturated 0.1 m KOH with a scan rate of 50 mV s^−1^. LSV tests were performed using the RDE in O_2_‐saturated 0.1 m KOH solution at a potential range from 0.2 to −1 V (vs Ag/AgCl) with a sweep rate of 10 mV s^−1^ at 1600 rpm. Long‐term stability test was carried out in O_2_‐saturated solution for 10 h. Methanol crossover measurement was conducted when 1.0 m methanol was injected into the electrolyte at 400 s.

The ORR kinetic for Fe_0.25_Co_0.75_/NC was carried out by the RDE method. The electron transfer number was calculated from the K–L equation at various potentials as follows

(2) $$\frac{1}{J}=\frac{1}{J_{L}}+\frac{1}{J_{K}}=\frac{1}{B\omega^{1/2}}+\frac{1}{J_{K}}$$

(3) $$B=0.62nFC_{0}{\left(D_{0}\right)}^{2/3}v^{-1/6}$$

where *J* is the measured current density; *J*_L_ and *J*_K_ are the diffusion limiting and kinetic current densities, respectively; *ω* is the angular velocity; *n* is the electron transfer number; *F* is the Faraday constant (96 485 C mol^–1^); *C*_0_ is the bulk concentration of O_2_ (1.2 × 10^–3^ mol L^–1^); *D*_0_ is the diffusion coefficient of O_2_ (1.9 × 10^–5^ cm^2^ s^–1^); *ν* is the kinematic viscosity of the electrolyte (0.01 cm^2^ s^–1^).

To further clarify the ORR mechanism of the resultant catalysts, the transfer number of electrons (*n*) and the yield of H_2_O_2_ during the ORR reaction were estimated based on the rotating ring‐disk electrode (RRDE) according to Equations (4) and (5)

(4) $$H_{2}O_{2}\left(\%\right)=200\times \frac{I_{R}/N}{I_{D}+I_{R}/N}$$

(5) $$N=4\times \frac{I_{D}}{I_{D}+I_{R}/N}$$

where *I*_R_ and *I*_D_ represent the current at the ring and disk, respectively; *N* is the current collecting efficiency of the Pt ring and determined to be 0.37.

The TOF value was calculated based on Equation (6)^[^ ^57^ ^]^

(6) $$\mathrm{TOF}=\left(J\times A\right)/\left(4\times F\times n\right)$$

where *J* is the current density at a given potential (0.8 V), *A* is the surface area of the glassy carbon electrode (0.19625 cm^2^), the number of 4 represents 4 electrons mol^−1^ of O_2_, *F* is the Faraday constant (96 485.3 C mol^−1^), and *n* stands for the number of Fe and Co atoms in samples.

### DFT Calculations

The theoretical evaluations were conducted using Vienna Ab initio Simulation Package (VASP) code.^[^ ^58^ ^]^ The core electrons were treated using projected augmented wave pseudopotentials,^[^ ^59^, ^60^ ^]^ while the electron–ion interactions and electronic exchange correlations were described by the formulations of Perdew–Burke–Ernzerhof^[^ ^61^ ^]^ and ultra‐soft potentials within the generalized gradient approximation.^[^ ^62^ ^]^ The Kohn–Sham equations were approached using a plane‐wave basis set at a cutoff energy of 500 eV, with the convergence thresholds being 10^–5^ eV cell^−1^ in energy and 0.015 eV A^−1^ in force, respectively. In the Fe_0.2_Co_0.8_ (110) (Fe:1, Co:4), Fe_0.25_Co_0.75_ (110) (Fe:1, Co:3), Fe_0.3_Co_0.7_ (110) (Fe:3, Co:7), and monometallic counterparts slab models, slabs were adopted with hexa‐layers, which contained eight atoms per layer. A large vacuum gap of 15 Å along the *z*‐axis was employed to prevent possible interlayer interactions. The p (2 × 2) supercells of Fe*_x_* Co*_y_* and monometallic counterparts were examined with a 3 × 3 × 1 k‐Point mesh. The adsorbed species were adhered to the top of the active sites (on the relaxed free surface) at a certain distance to undergo calculation.

The adsorption energy was then calculated using the following formula^[^ ^63^, ^64^ ^]^

(7) $$E_{\mathrm{ads}}=E_{\mathrm{sub}+\mathrm{adatom}}-E_{\mathrm{sub}}-E_{\mathrm{adatom}}$$

where *E*_sub+adatom_, *E*_sub_, and *E*_adatom_ refer to the total energy of the FeCo alloy and adsorbate, the energy of the FeCo substrate and the energy of the isolated adsorbate, respectively.

The ORR reaction free energies of each elementary step in alkaline media (pH = 13) was calculated according to the following two equations as well as introduction of computational hydrogen electrode approximation^[^ ^65^ ^]^

(8) ΔG=ΔH−TΔS

(9) $$\Delta H=\Delta E_{\mathrm{el}}+\Delta \mathrm{ZPE}+E_{\mathrm{pH}}+E_{\mathrm{thermal}}+PV$$

where ∆*E*_el_ indicates the electronic energy calculated via DFT methods, and ∆ZPE, *E*_pH_, *E*_thermal_ are corrections terms of zero‐point vibrations, pH variation and thermal contributions, respectively.^[^ ^66^, ^67^ ^]^

## Conflict of Interest

The authors declare no conflict of interest.

## Supporting information

Supporting information

## Data Availability

The data that support the findings of this study are available on request from the corresponding author. The data are not publicly available due to privacy or ethical restrictions.
