# Supplementary material for: Modulating the Electronic Structure of FeCo Nanoparticles in N‐Doped Mesoporous Carbon for Efficient Oxygen Reduction Reaction
Source: Adv Sci (Weinh). 2022 Mar 24;9(15):2200394. doi: 10.1002/advs.202200394 (PMC9130874; doi:10.1002/advs.202200394)
Supplement: Supplementary file 1 — Supporting information [file ADVS-9-2200394-s001.pdf]

Supporting information

Modulating the Electronic Structure of FeCo Nanoparticles in N-Doped Mesoporous Carbon for Efficient Oxygen Reduction Reaction

Guihua Zhu[a], Haoyu Yang[b], Ying Jiang[c], Ziqi Sun[b], Xiaopeng Li[a], Jianping Yang[a], Haifeng Wang[a], Rujia Zou[a], Wan Jiang[a], Pengpeng Qiu[a*], Wei Luo[a*]

[a]State Key Laboratory for Modification of Chemical Fibers and Polymer Materials, College of Materials Science and Engineering, Institute of Functional Materials, Donghua University, Shanghai 201620, China.

[b]School of Chemistry and Physics, Centre for Materials Science, Queensland University of Technology (QUT), Brisbane, QLD 4001, Australia.

[c]Materials Genome Institute, Shanghai University, Shanghai, 200444, PR China

Email of corresponding author: qiupengpeng@dhu.edu.cn, wluo@dhu.edu.cn

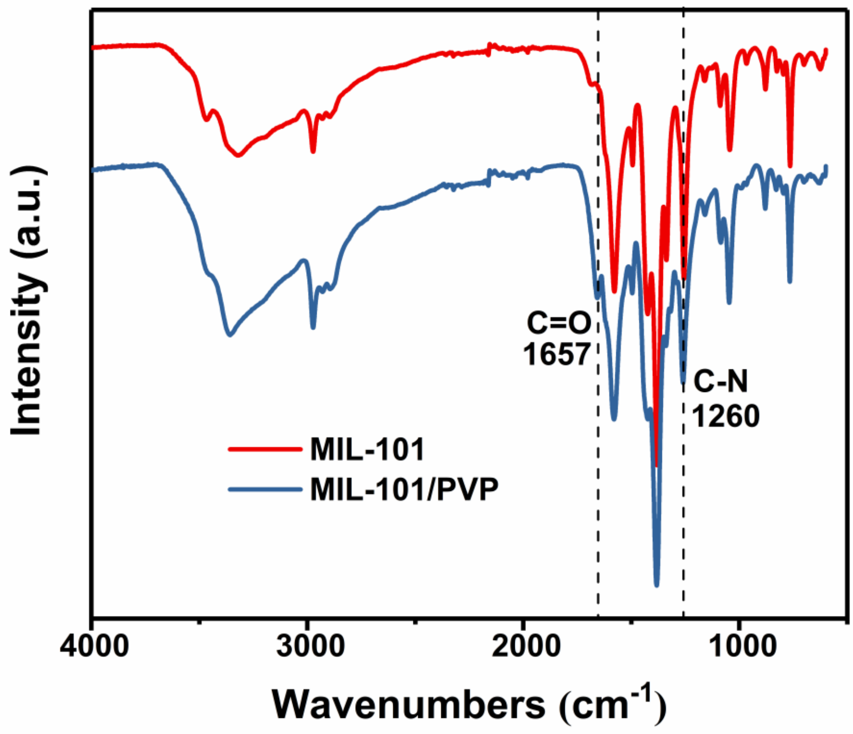

Fig. S1 FTIR spectrum of MIL-101 and MIL-101/PVP

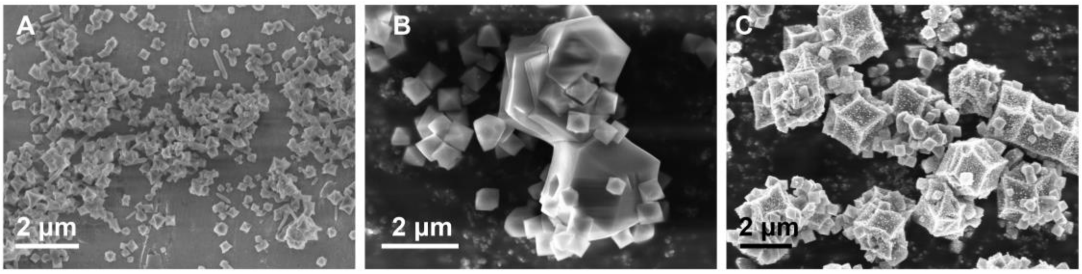

Fig. S2 SEM images of the precursor MIL-101 without PVP functionalization, corresponding MIL-101@ZIF-67, and FeCo/NC-800

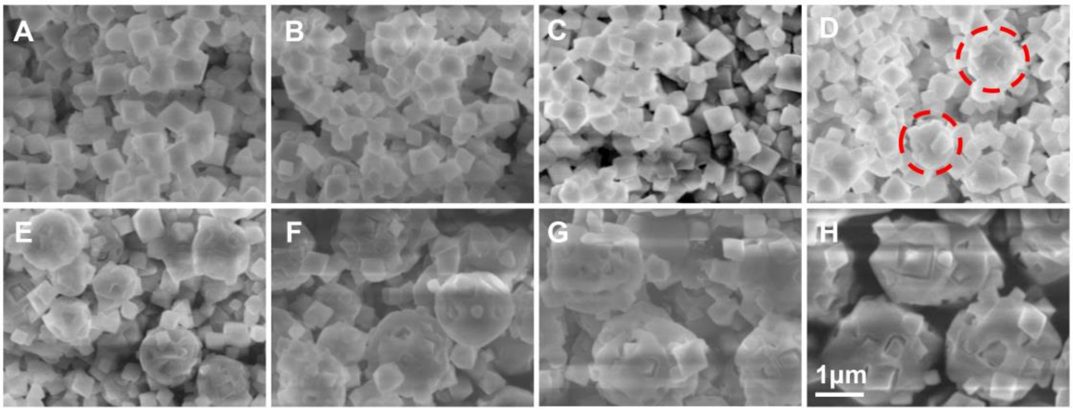

Fig. S3 SEM images of the intermediate product at the reaction time of 2 (A), 5 (B), 10 (C), 20 (D), 30 (E), 60 (F), 120 (G), and 240 (H) min.

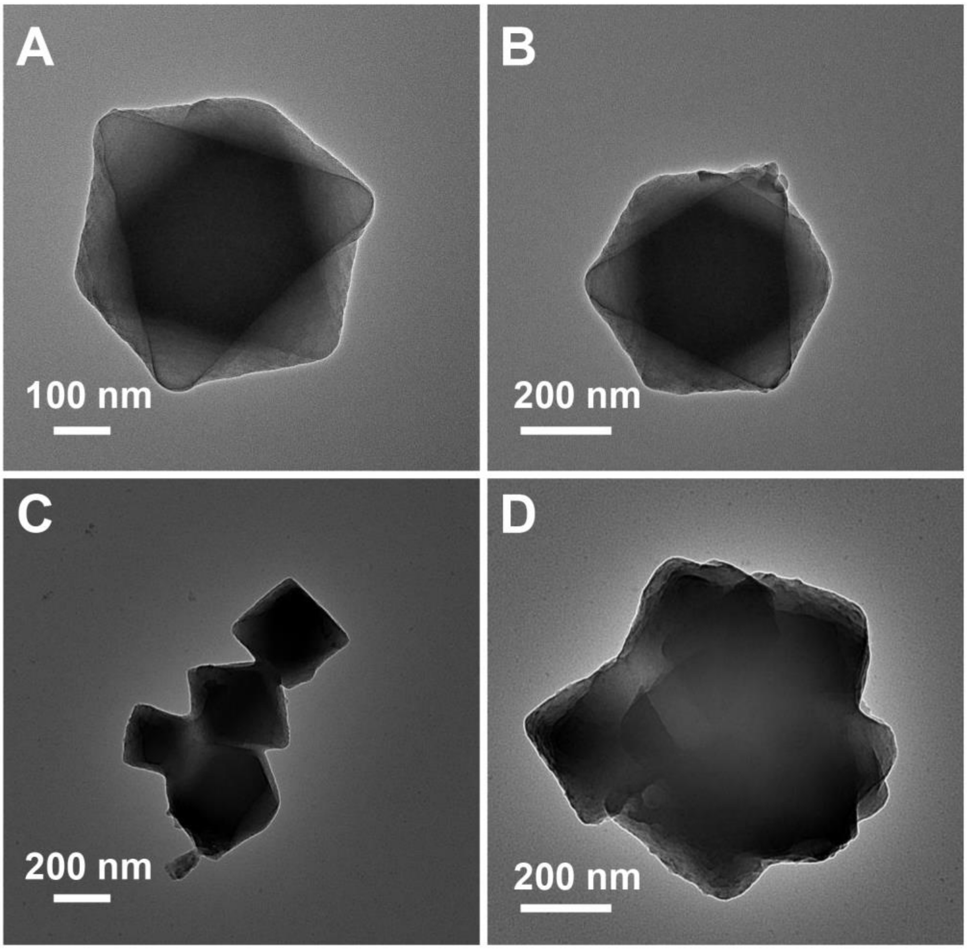

Fig. S4 TEM images of the intermediate product at the reaction time of 2 (A), 5 (B), 10 (C) and 20 (D) min.

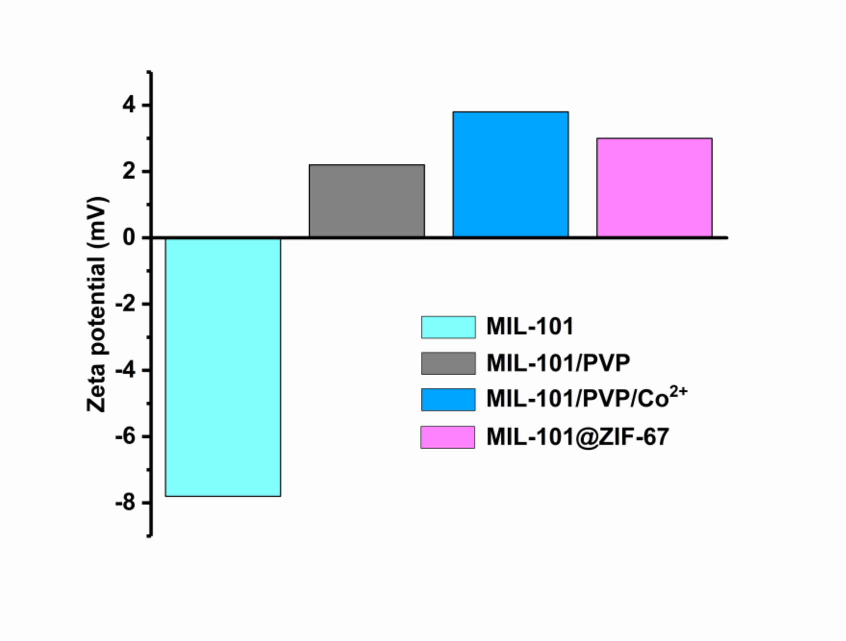

Fig. S5 Zeta potential of the sample during the reaction

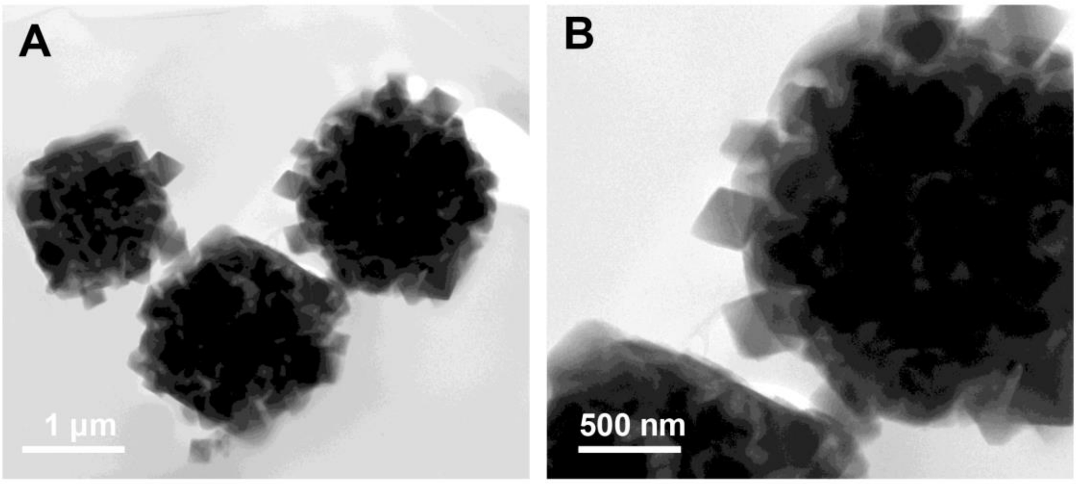

Fig. S6 TEM images of the precursor MIL-101@ZIF-67

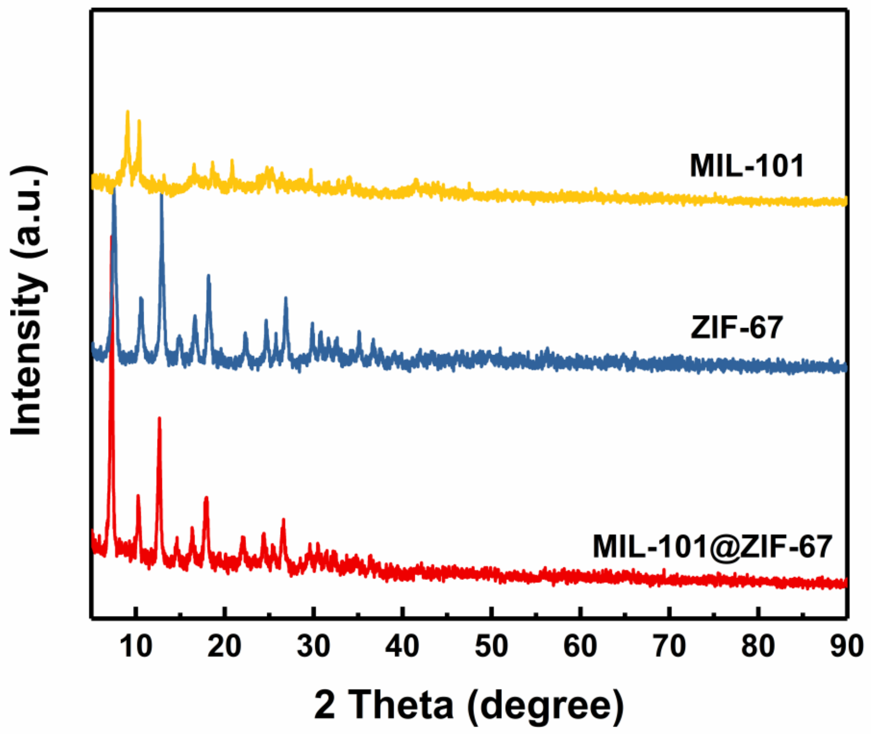

Fig. S7 XRD analysis of the MIL-101, ZIF-67, and MIL-101@ZIF-67

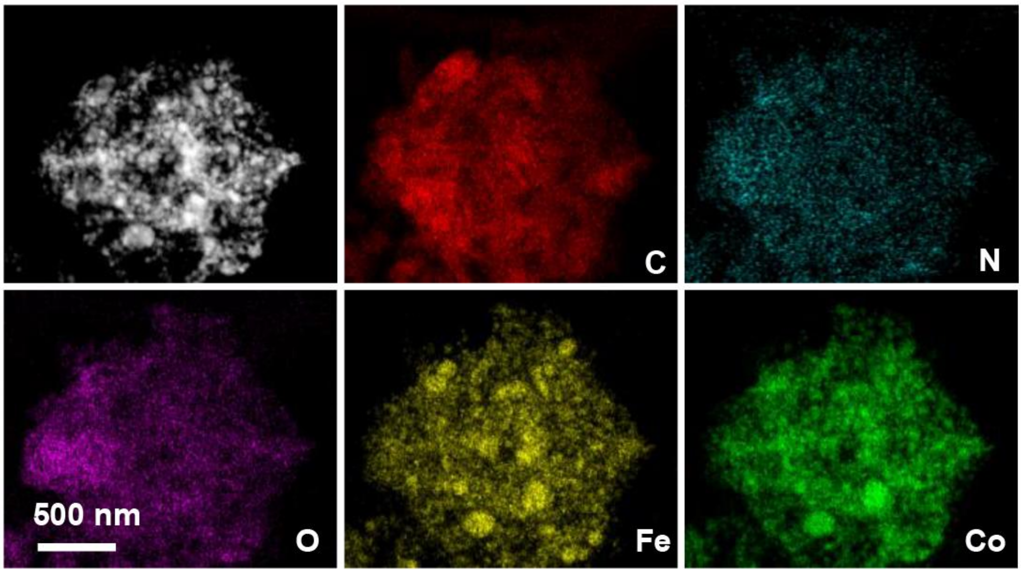

Fig. S8 EDS mapping of $Fe_{0.25}Co_{0.75}$/NC-800

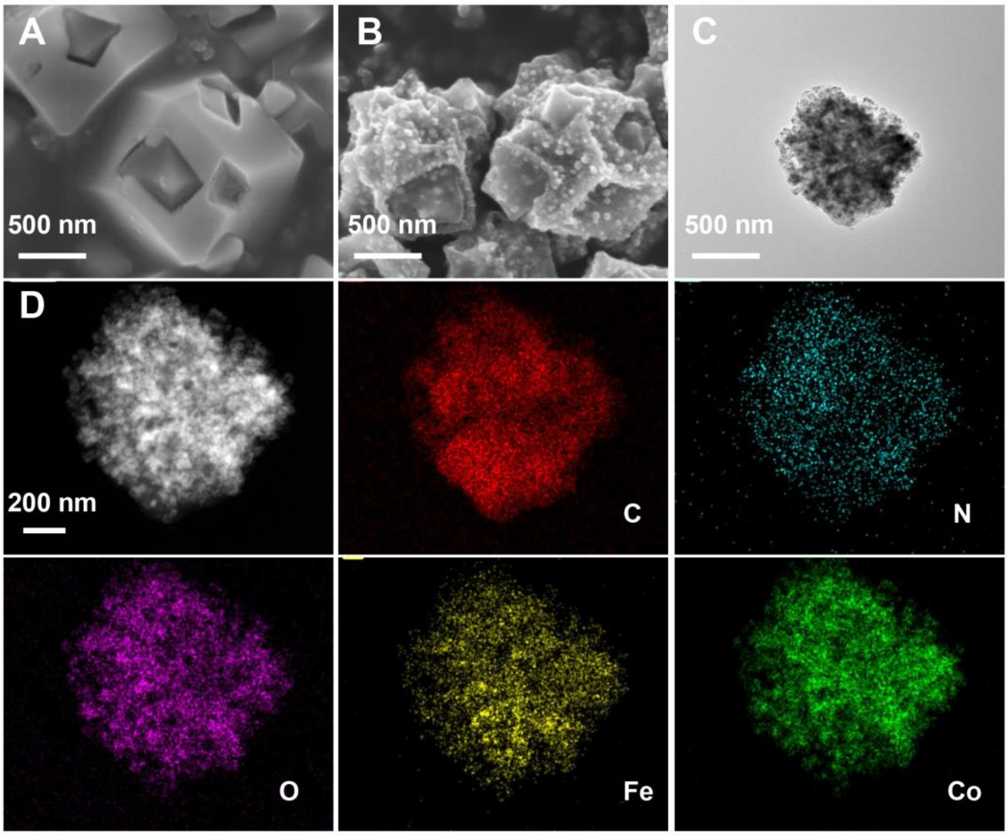

Fig. S9 SEM images, TEM images and EDS mapping of precursor and $Fe_{0.2}Co_{0.8}$/NC-800

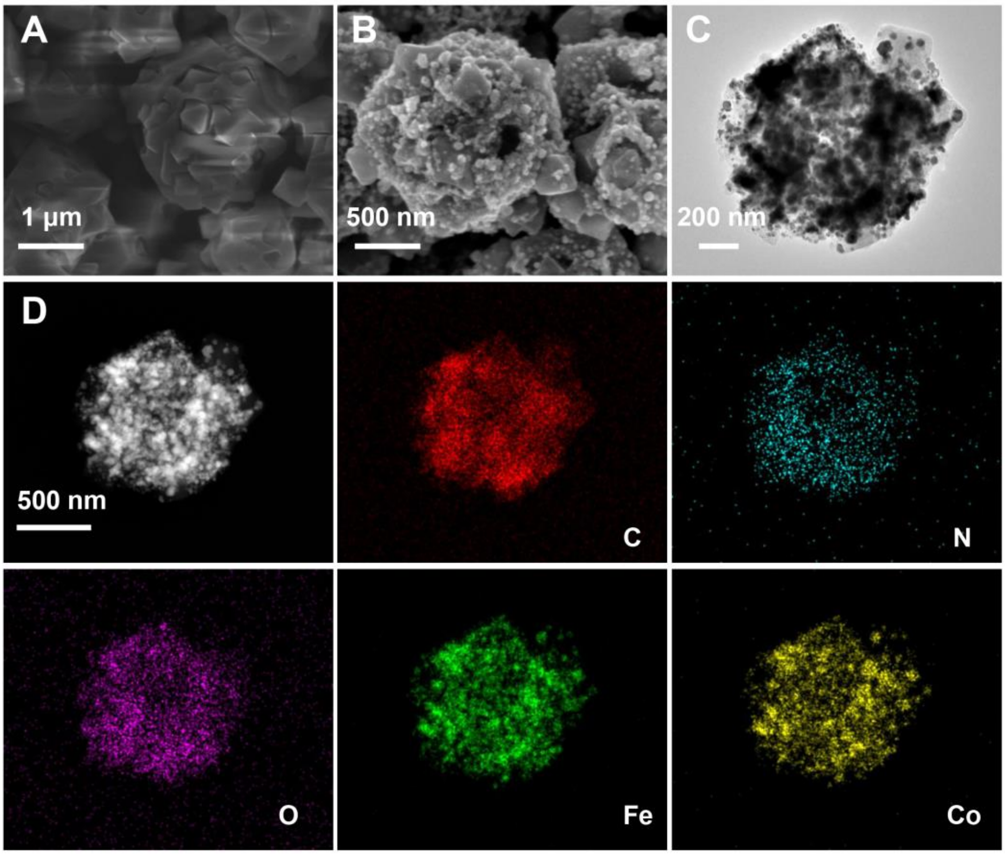

Fig. S10 SEM images, TEM images and EDS mapping of precursor and $Fe_{0.3}Co_{0.7}$/NC-800.

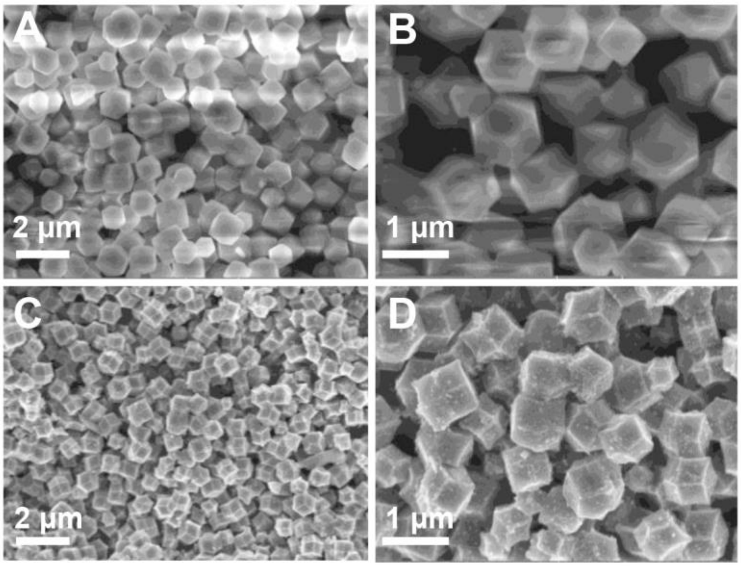

Fig. S11 SEM images of the (A, B) ZIF-67 and (C, D) Co/NC-800

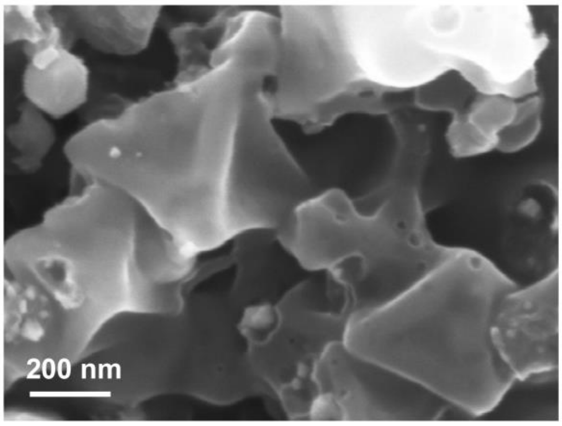

Fig. S12 SEM images of Fe/NC-800

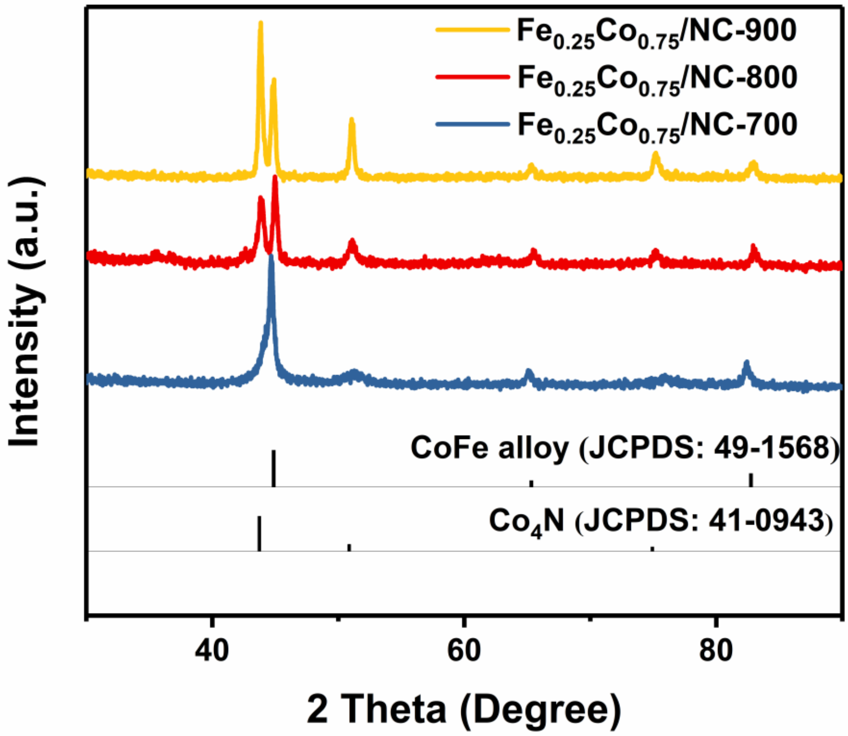

Fig. 13 XRD analysis of the $Fe_{0.25}Co_{0.75}$/NC catalyst calcined at different temperatures (700°C, 800°C, 900°C)

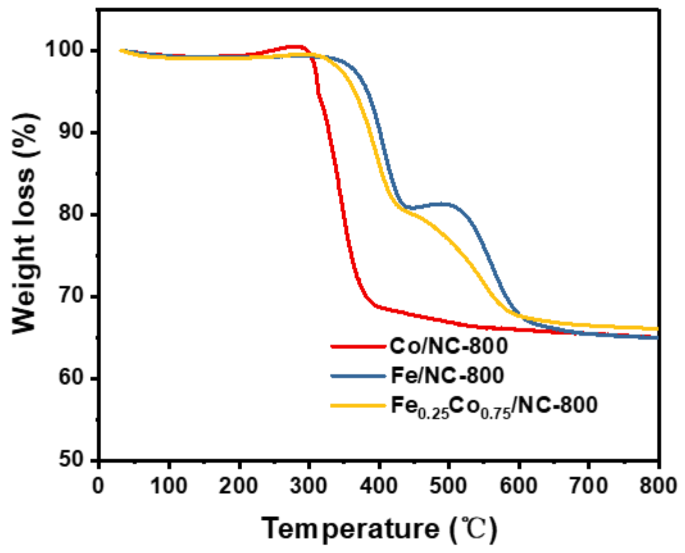

Fig. S14 TGA analysis of Fe/NC-800, Co/NC-800, and $Fe_{0.25}Co_{0.75}$/NC-800 electrocatalysts.

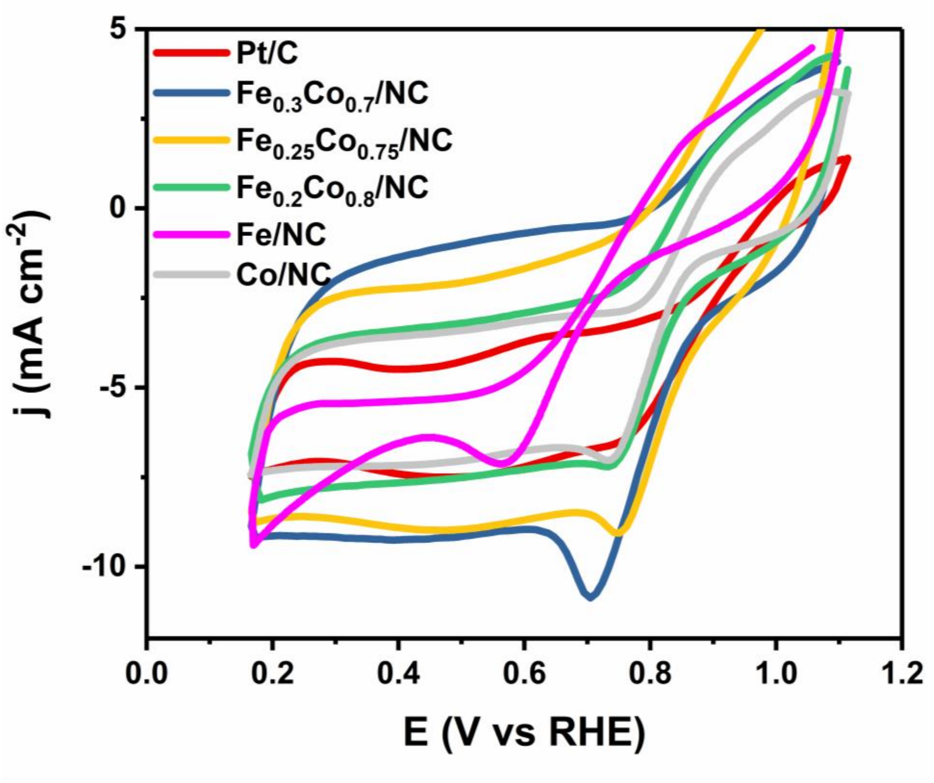

Fig. S15 CV of the as-synthesized electrocatalysts in O_2-saturated 0.1 M NaOH solution

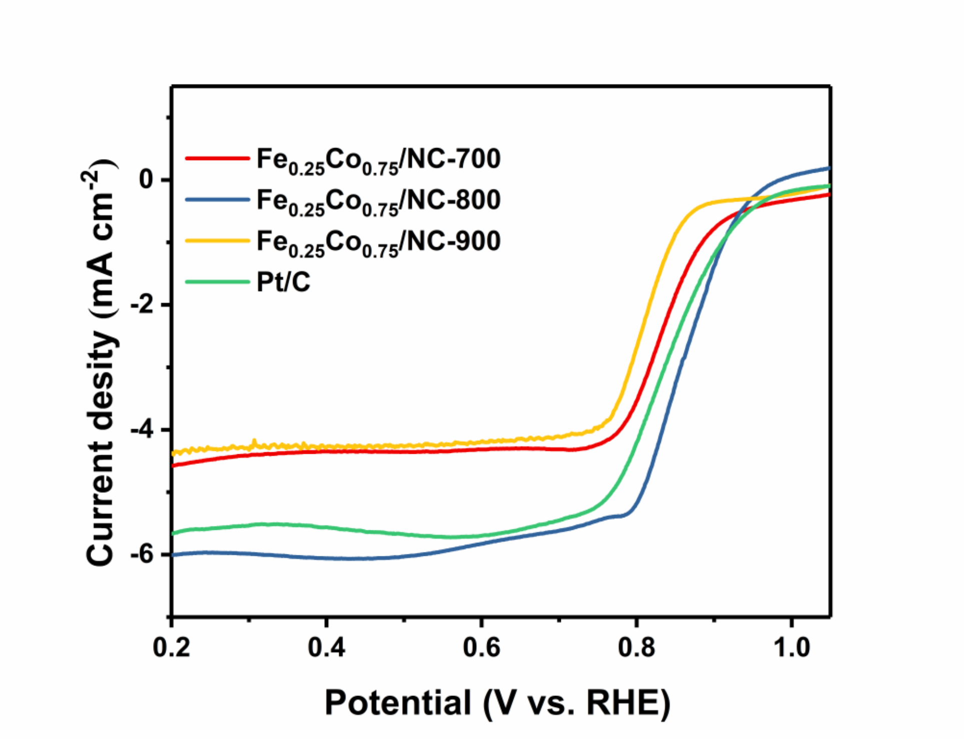

Fig. S16 Polarization curves of the ORR process for $Fe_{0.25}Co_{0.75}$/NC electrocatalyst with different pyrolysis temperature and commercial Pt/C in 0.1 M NaOH

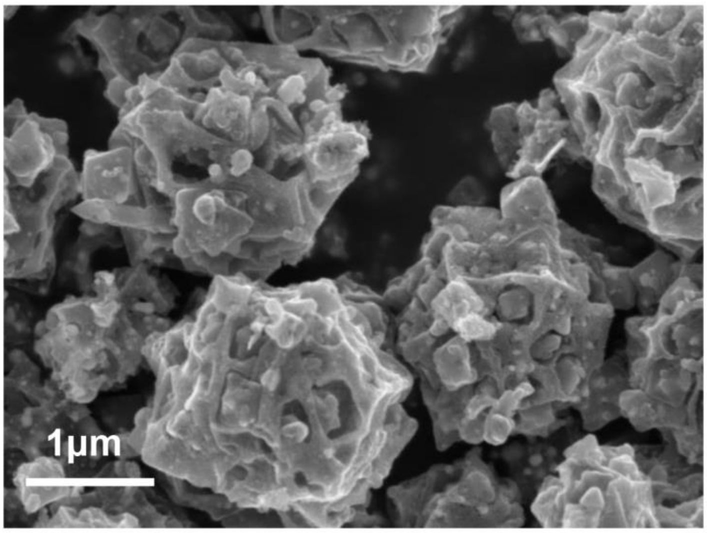

Fig. S17 SEM image of $Fe_{0.25}Co_{0.75}$/NC-900 electrocatalyst.

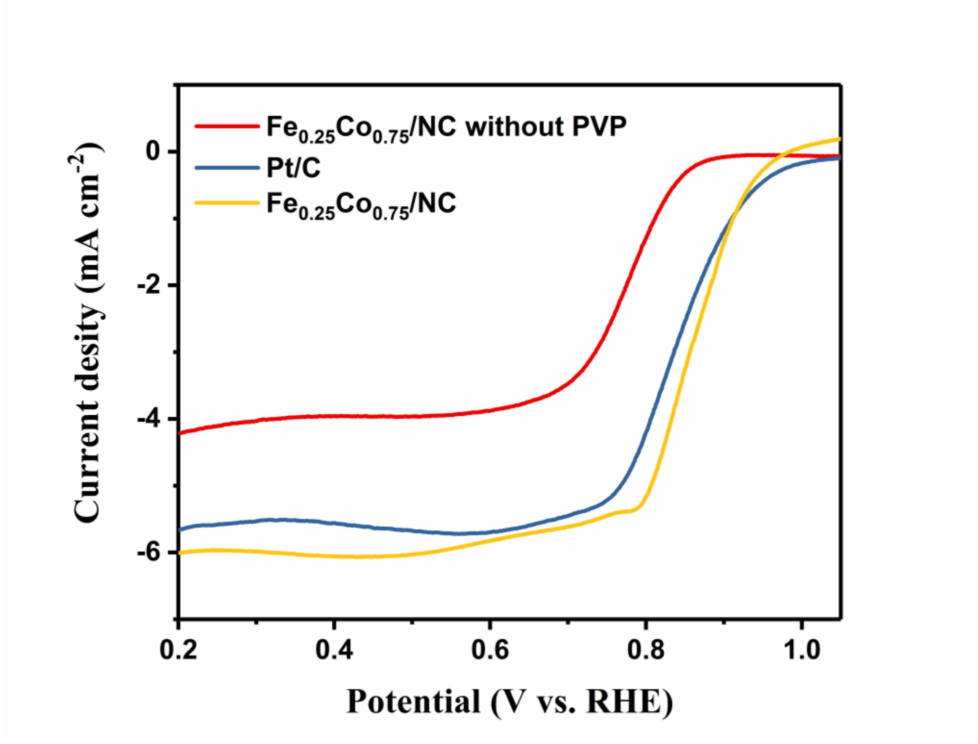

Fig. S18 Polarization curves of the ORR process for $Fe_{0.25}Co_{0.75}$/NC electrocatalyst and contrast without PVP function in 0.1 M NaOH

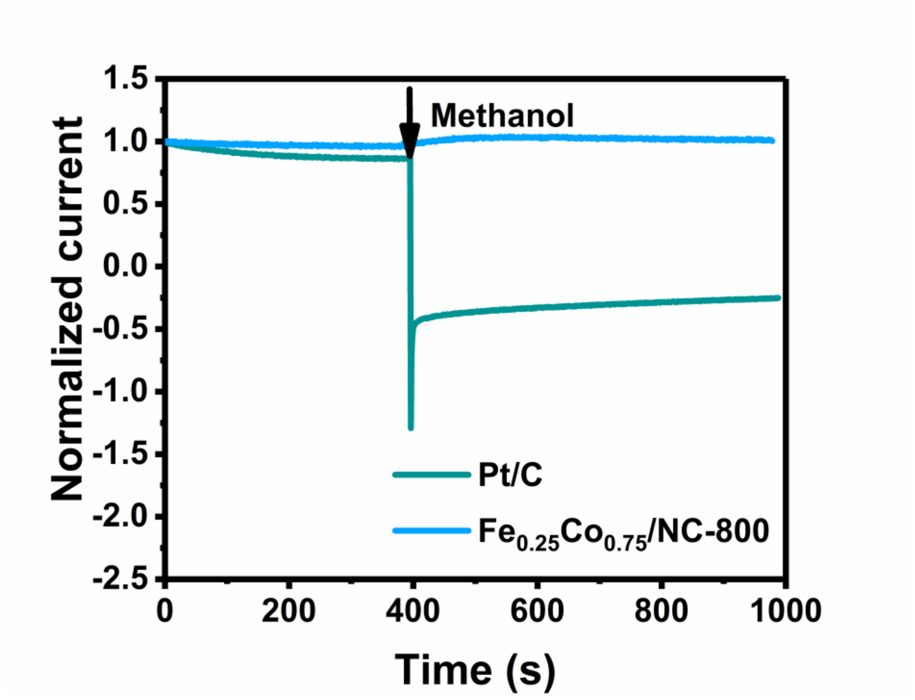

Fig. S19 Chronoamperometric response at 0.6 V vs RHE after the injection of 1M methanol into 0.1 M KOH solution.

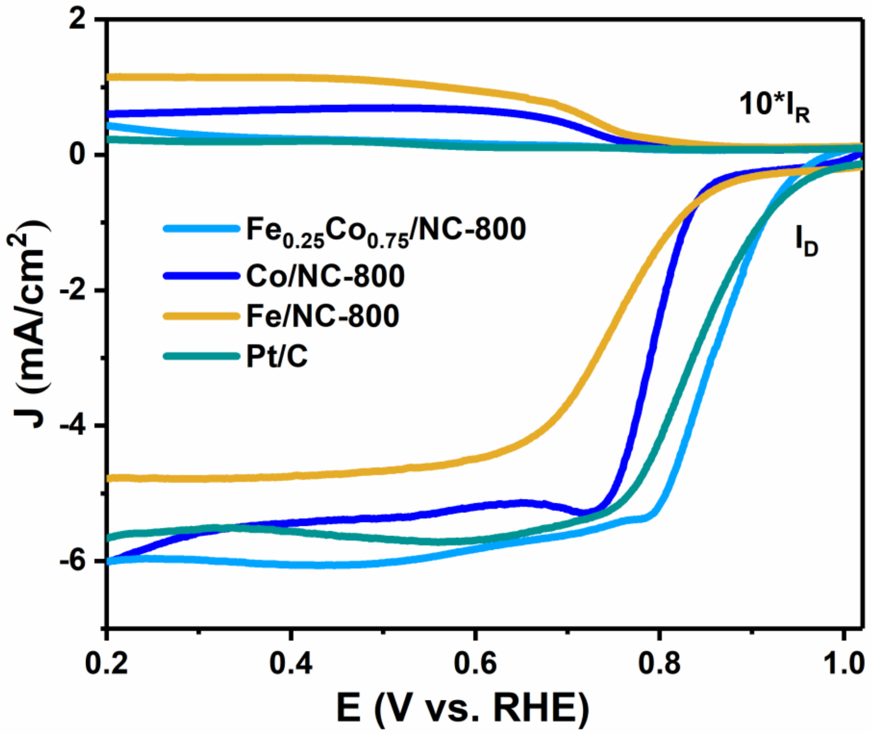

Fig. S20 RRDE curves of Fe0.25Co0.75/NC-800 and monometallic counterparts in 0.1 M O2-saturated KOH solution at 1600 rpm. For clear observation, the current collected from the ring was 10 times enlarged.

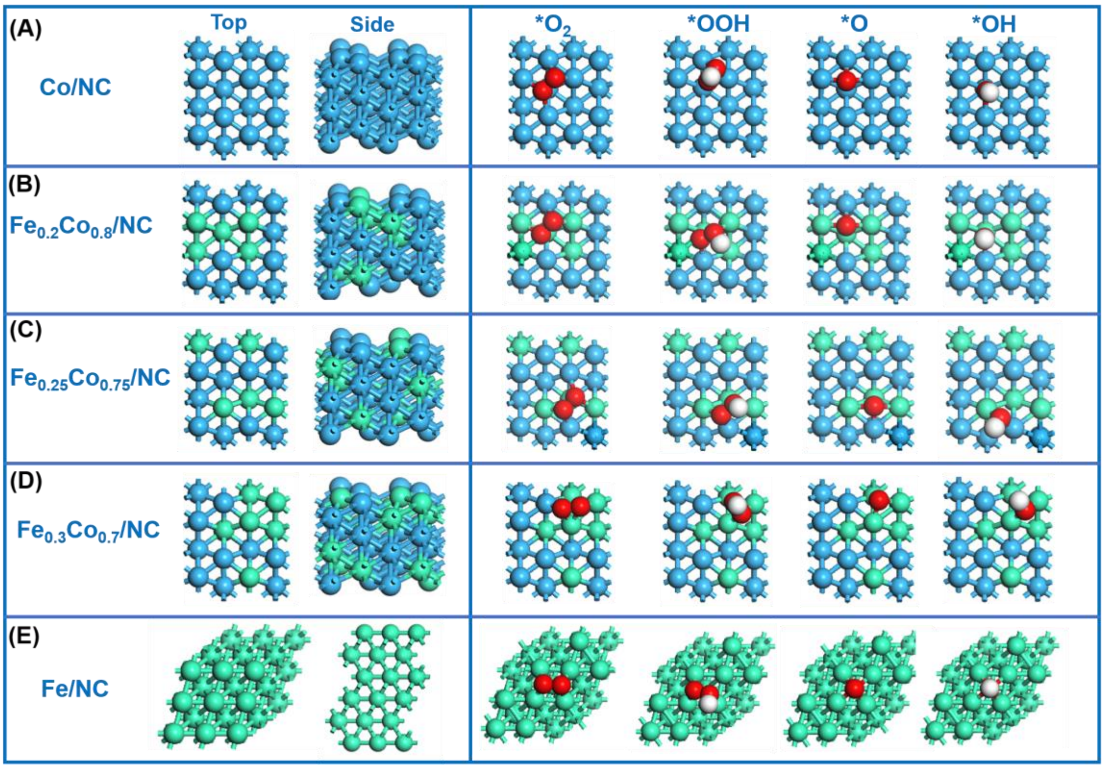

Fig. S21 Slab model (left) and ORR intermediates adsorption (right) ($*O_2$, *OOH, *O, and *OH) on electrocatalyst's surface (blue, green, red, and white spheres represent cobalt, iron, oxygen and hydrogen, respectively) (A) Co/NC (B) $Fe_{0.2}Co_{0.8}$/NC (C) $Fe_{0.25}Co_{0.75}$/NC (D) $Fe_{0.3}Co_{0.7}$/NC and (E) Fe/NC

Table S1. The element composition of the electrocatalysts obtained by ICP-OES measurements.

Samples	Fe content (mg/g)	Co content (mg/g)
$Fe_{0.2}Co_{0.8}$/NC	108.6	499.2
$Fe_{0.25}Co_{0.75}$/NC	125.7	372.4

$Fe_{0.3}Co_{0.7}$/NC	112.8	304.1

Table S2. BET surface areas, micropore surface area, total pore volumes, pore sizes, and average pore diameters of as-synthesized catalysts.

Sample	BET surface area (m^2g^{-1})	Micropore surface area (m^2g^{-1})	Total pore volume (cm^3g^{-1})	Pore size (nm)
$Fe_{0.25}Co_{0.75}$/NC	223.53	27.56	0.32	3.828
Fe/NC	248.98	66.44	0.32	3.834
Co/NC	274.15	65.81	0.36	3.825

Table S3. Different atomic percentages of the near-surface regions of as-synthesized catalysts tested by XPS

Samples	C (atom %)	N (atom %)	O (atom %)	Fe (atom %)	Co (atom %)
Fe/NC	91.25	3.39	4.77	0.59	/
Co/NC	84.81	5.84	7.2	/	2.14
$Fe_{0.2}Co_{0.8}$/NC	77.66	5.24	13.38	0.28	3.43
$Fe_{0.25}Co_{0.75}$/NC	81.32	5.31	10.21	0.52	2.64
$Fe_{0.3}Co_{0.7}$/NC	85.08	4.06	8.63	0.16	2.07

Table S4. Content of pyridinic-N, graphitic-N, pyrrolic-N and oxidic-N of

as-synthesized catalysts evaluated by XPS

Samples	Pyridinic-N content (%)	Pyrrolic-N content (%)	Graphitic-N content (%)	Oxidized-N content (%)
Fe/NC	13.63	11.7	57.4	17.27
Co/NC	26.86	26.13	28.68	18.33
$Fe_{0.2}Co_{0.8}/NC$	27.8	18.56	31.01	22.63
$Fe_{0.25}Co_{0.75}/NC$	15.54	34.23	26.69	23.53
$Fe_{0.3}Co_{0.7}/NC$	17.73	19.03	32.92	30.31

Table S5. Comparison of ORR performances with previously reported electrocatalysts.

Catalysts	$E_{1/2}$ (V vs RHE)	Limiting current density (mA cm^{-2})	Reference
$Fe_{0.25}Co_{0.75}/NC$	0.860	6.010	This work
Fcc-FeCo/NCNT	0.8	5.000	[1]
$Fe_{0.5}Ni_{0.5}$@N-GR	0.83	4.050	[2]
MCCF/NiMn-MOFs	0.73	5.600	[3]
FeCo@MNC	0.860	5.300	[4]
FeCo/NC	0.848	5.700	[5]
$Fe_{1.2}Co$@NC/NCNTs	0.820	5.650	[6]
Pd/FeCo	0.840	6.220	[7]
FeCo/N-DNC	0.810	6.150	[8]

FeCo–NC_{ps}	0.845	5.730	[9]
FeCo/NPC	0.810	4.850	[10]
FeCo-N-C	0.838	7.660	[11]
CoZn−N−C	0.834	5.420	[12]
FeCo-NCNFs-800	0.817	5.370	[13]
FeCo/Co_2P@NPCF	0.790	4.980	[14]

References

[1] R. Nandan, P. Pandey, A. Gautam, O. Y. Bisen, K. Chattopadhyay, M.-M. Titirici,K. K. Nanda, *ACS Appl. Mater. Interfaces* **2021**, *13*, 3771.

[2] P. Liu, D. Gao, W. Xiao, L. Ma, K. Sun, P. Xi, D. Xue,J. Wang, *Adv. Funct. Mater.* **2018**, *28*, 1706928.

[3] W. Cheng, X. F. Lu, D. Luan,X. W. Lou, *Angew. Chem. Int. Ed.* **2020**, *59*, 18234.

[4] C. Li, M. Wu,R. Liu, *Appl. Catal. B: Environ.* **2019,** *244*, 150.

[5] L. Cui, M. Chen, G. Huo, X.-Z. Fu,J.-L. Luo, *Chem. Eng. J.* **2020,** *395*, 125158.

[6] S. Li, W. Chen, H. Pan, Y. Cao, Z. Jiang, X. Tian, X. Hao, T. Maiyalagan,Z.-J. Jiang, *ACS Sustain. Chem. Eng.* **2019,** *7*, 8530.

[7] F. Pan, Z. Li, Z. Yang, Q. Ma, M. Wang, H. Wang, M. Olszta, G. Wang, Z. Feng,Y. Du, *Adv. Energy Mater.* **2021,** 11, 2002204.

[8] G. Fu, Y. Liu, Y. Chen, Y. Tang, J. B. Goodenough,J.-M. Lee, *Nanoscale* **2018,** *10*, 19937.

[9] J. Liu, T. He, Q. Wang, Z. Zhou, Y. Zhang, H. Wu, Q. Li, J. Zheng, Z. Sun,Y. Lei, *J Mater. Chem. A* **2019,** *7*, 12451.

[10] R. Gao, Y. Yin, F. Niu, A. Wang, S. Li, H. Dong, S. Yang, *ChemElectroChem* **2019,** *6*, 1824.

[11] X. Jin, Y. Xie, L. Wang,J. Huang, *ChemElectroChem* **2020,** *7*, 865.

[12] J.-L. Mi, J.-H. Liang, L.-P. Yang, B. Wu, L. Liu, *Chem. Mater.* **2019,** *31*, 8864.

[13] L. Yang, S. Feng, G. Xu, B. Wei, L. Zhang, *ACS Sustain. Chem. Eng.* **2019,** *7*, 5462.

[14] Q. Shi, Q. Liu, Y. Ma, Z. Fang, Z. Liang, G. Shao, B. Tang, W. Yang, L. Qin,X. Fang, *Adv. Energy Mater.* **2020,** *10*, 1903854.
